# HPLC-DAD-MS Identification and Quantification of Phenolic Components in Japanese Knotweed and American Pokeweed Extracts and Their Phytotoxic Effect on Seed Germination

**DOI:** 10.3390/plants11223053

**Published:** 2022-11-11

**Authors:** Maja Mikulic-Petkovsek, Robert Veberic, Metka Hudina, Eva Misic

**Affiliations:** Chair for Fruit Growing, Viticulture and Vegetable Growing, Department of Agronomy, Biotechnical Faculty, University of Ljubljana, Jamnikarjeva 101, SI-1000 Ljubljana, Slovenia

**Keywords:** invasive alien plant species (IAPS), Japanese knotweed, American pokeweed, phenolic compounds, HPLC-MS, phytotoxic effect, germination

## Abstract

We performed a detailed HPLC-MSn analysis of the phenolic compounds from the extracts of two invasive alien plant species (IAPS): Japanese knotweed (*Fallopia japonica* (Houtt.) Ronse Decr.) and American pokeweed (*Phytolacca americana* L.). The major phenolic groups were hydroxycinnamic acids and flavanols in Japanese knotweed (J. knotweed) and flavonols, hydroxycinnamic acids, and stilbenes in American pokeweed (A. pokeweed). We investigated the influence of solvent type and extraction time on the extraction efficiency of the phenolic compounds. The solvent 80% methanol had a higher polyphenolic extraction efficiency than water, since 14.5 times more flavonols and 2.3 times more stilbenes were extracted from J. knotweed and 5.2 times more flavonols and 2.6 times more stilbenes were extracted from A. pokeweed. In contrast, with water, we obtained a 52% higher hydroxycinnamic acids (HCA) content from J. knotweed. Hydroxycinnamic acids were best extracted in water after 24 h, flavanols after 12 h, stilbenes between 12 and 24 h, and flavonol glycosides after 48 h of extraction. We also tested the allelopathic effect of the aqueous extract of A. pokeweed and J. knotweed on seed germination and shoot and root growth of perennial ryegrass. The results showed that the water extract of J. knotweed resulted in 38 to 48% lower seed germination of perennial ryegrass, and the extract of A. pokeweed resulted in 83 to 90% lower seed germination. The phytotoxic effect of the extract of J. knotweed and A. pokeweed was also reflected in a characteristic reduced growth of shoots and roots of perennial ryegrass. The phytotoxic action of IAPS could also be applied for beneficial purposes, since this would be an effective strategy for their control and a reduction of their spread in the environment.

## 1. Introduction

Some invasive alien plant species (IAPS) nowadays pose an increasing threat because they negatively impact the environment, reduce biodiversity, significantly alter ecosystem functioning and negatively affect human health [1,2]. Invasive plants have unique characteristics that make them more competitive than native flora. They are characterized by rapid growth, rapid reproduction (sexually through seeds and asexually through vegetative parts) and dispersal [3].

One of the survival strategies of IAPS is the synthesis of allelochemicals that can directly or indirectly affect the growth of native plants [1]. Plants release allelochemical compounds into the environment in different ways, which affects the growth and development of other plants [4]. Kalisz et al. [5] estimated that most invasive plant species contain allelochemicals, suggesting that allelopathy is an important property of invasive plants, and it is considered to be a fundamental mechanism by which IAPS displace native species [3]. Various studies have shown that IAPS have a distinct ecological adaptability, which is mainly manifested in high genetic diversity, greater resistance to stressors and broad ecological niches [2].

Most authors include allelochemicals primarily as secondary metabolites that plants release to the environment in various ways [6]. Disruption of photosynthesis is one of the most common physiological effects of allelochemicals. In addition, allelochemical compounds can affect cell respiration, mineral uptake, cell division and so on [4,6,7]. However, their mode of action is often not well understood [8]. Allelochemicals are released into the environment through root exudates, volatilization or leaching from plants, decay of donor plant residues, and seed exudates [1,7].

Secondary metabolites are often synthesized by plants in response to adverse conditions or at certain stages of development. They arise from basic metabolic pathways for the synthesis of carbohydrates, amino acids and fatty acids. Secondary metabolites are often species-specific and characteristic of individual taxonomic groups [6]. Most compounds that can act as allelochemicals belong to the phenolics group [9].

The success of extraction of plant metabolites depends on both the type of extraction and the extraction conditions (particle size, type of solvent, temperature, time, ratio of solvent to plant material, etc.) [10,11,12]. Although various extraction techniques are known, including enzyme-assisted ultrasonic extraction, pulsed electric field extraction, microwave liquid extraction etc., solvent extraction is still the most commonly used [13,14,15]. The advantages of this process include its ease of use and the fact that no additional equipment is required. An important step in the extraction process is the selection of an appropriate solvent that will determine the profile of the bioactive compounds. Since secondary metabolites have different chemical compositions, e.g., different number of hydroxyl groups, different attached functional groups that differ in number and position in the compound, their solubility is also different. Solubility also depends on hydroxylation, methylation and glycolysis [16,17,18]. In addition, it should be taken into account that higher extraction temperatures contribute to the decomposition of some phenolic compounds. The most commonly used solvents for the extraction of polyphenolic substances are ethanol, methanol, ethyl acetate, acetone, chloroform and various combinations of these solvents with water [10,17]. It is certainly best to use water as the solvent to balance economy and an environmentally friendly, natural approach [19].

More and more research is being conducted towards the possible beneficial use of invasive plant species and, consequently, their reduction in the environment. Reviewing the literature, we found that studies in the field of monitoring the efficiency of extraction of phenolic compounds from the plant parts of Japanese knotweed (*Fallopia japonica*) and American pokeweed (*Phytolacca americana*) are very scarce. Our aim was therefore to determine which solvent is best for extracting phenolic compounds from these species and how the extraction time affects the extraction efficiency from these plants. The second aim was to identify the phenolic compounds in these IAPS extracts (J. knotweed and A. americana) and analyze their content. An additional goal of this study was to determine whether a water extract of American pokeweed and Japanese knotweed can affect the germination of perennial ryegrass (*Lolium perenne* L.) seeds. The parameters we monitored were the percentage of germinating seeds, the length of the root and the length of the shoot. We wanted to investigate with this experiment whether extracts of selected invasive plant species could be used as a potential source for inhibiting the growth of some plants (e.g., weeds).

## 2. Results and Discussion

### 2.1. Phytochemical Analysis of Japanese Knotweed and American Pokeweed Extract and Phytotoxic Activity of Their Extracts

We identified 62 different phenolic compounds from seven different phenolic groups in J. knotweed extract (Table 1, Supplementary Table S1). Extraction of individual polyphenolic compounds from J. knotweed with methanol was more successful for most polyphenolics because a higher concentration of polyphenolic compounds was extracted with methanol. Only in the extraction of hydroxybenzoic acids and hydroxycinnamic acids from J. knotweed, water proved to be the best solvent (Table 1).

The major phenolic groups in the water extract of J. knotweed were hydroxycinnamic acids (53.8% of the total analyzed phenolic compounds, TAP) and flavanols 40.6% TAP, followed by stilbenes (2.8% TAP), flavonols (2.3% TAP), while hydroxybenzoic acid (0.51% TAP), flavones (0.01% TAP) and quinones (0.001% TAP) were present in very small amounts. Among the hydroxycinnamic acids, caftaric acid 1 (25,640.47 µg/g DW) ranked first, its content accounting for almost 90% of the total HCAs (Table 1).

Caftaric acid (caffeoyl tartaric acid) was confirmed based on the deprotonated molecular ion [M-H] at *m*/*z* 311 and the fragment ion generated at *m*/*z* 179, corresponding to the loss of the tartaric acid moiety (*m*/*z* 132 u) and the caffeoyl moiety (*m*/*z* 162 u) (Supplementary Table S1). Lachowicz et al. [20] also reported the presence of caftaric acid in J. knotweed. We confirmed two caftaric acids in J. knotweed (Table 1), and they were probably the *cis-* and *trans*-forms. In addition to them, we also analyzed the presence of other phenolic acids, with the largest number being quinic acid components, which were bound with caffeoyl, dicaffeoyl, feruloyl and coumaroyl structures. Representatives of hydroxybenzoic acids were detected only in the aqueous extract. From the group of flavanols, many procyanidins, i.e., dimers, trimer and tetramers, were found in the J. knotweed extract (Table 1). In addition, there were also two derivatives of catechin and epicatechin. There was a fairly large group of flavonol glycosides in J. knotweed. Nine quercetin derivatives, three kaempferol derivatives and one myricetin and isorhamnetin derivative were thus identified. The major flavonol was quercetin-3-rhamnoside (14,636.7 µg/g DW). We thus obtained contents of individual quercetin glycosides from 0.13 to 14,636.7 µg/g DW of J. knotweed with 80% MeOH and with water from 0.03 to 951.59 µg/g DW (Table 1). In the stilbenes group, we found seven representatives (two resveratrolosides, two piceides, astringin and two piceatannol hexosides) and in the quinones group only emodin hexoside (Table 1).

It can be seen from Table 2 that both water extracts prepared from J. knotweed had an inhibitory effect on the germination of the perennial ryegrass seeds. When watering the seeds with a water extract concentration of 1:0.2 g/mL, the germination of ryegrass decreased by 47% and with a water extract concentration of 2:0.15 g/mL by 37.5%. However, there were no significant differences in the germination percentage of seeds between the prepared concentrations of the extract. The treatment with the extract of J. knotweed also resulted in characteristically shorter shoots and roots of the perennial ryegrass. Thus, for example, the extract of concentration 1 resulted in 14.7 mm shorter shoots and 19.54 mm shorter roots of perennial ryegrass compared to the control (Table 2).

A similar inhibitory effect on the growth of sprouts was also observed with the aqueous extract of J. knotweed at concentration 2. Previous research has indicated similar effects of knotweed. It has been found that root extracts of different J. knotweed species effectively inhibit the growth of white mustard [21]. Šoln et al. reported [22] on the negative effect of J. knotweed extract on the growth and development of radish roots.

In samples of American pokeweed, we identified 34 compounds from five phenolic groups (hydroxycinnamic acids, flavanols, flavones, flavonols, and stilbenes) (Table 3, Supplementary Table S2). In the methanol extract of A. pokeweed we detected five betalain derivatives, the total content of which was 1200 µg/g DW. They were not present in the aqueous extract. Marinas et al. [23] also determined betalains in methanol extracts of the fruits and leaves of A. pokeweed. In the methanol extract, we found 1.8 times higher content of flavone, 5.2 times higher content of total flavonols and 2.6 times higher content of total stilbenes compared to the aqueous extract. There were no significant differences in the content of total flavanols and hydroxycinnamic acid derivatives between the two extracts of A. pokeweed. In the group of hydroxycinnamic acids, the most important substance was 4-caffeoylquinic acid (4-CQA), which accounted for more than half of the total HCA content. In A. pokeweed, the content of 4-CQA was determined to be 1248.71 µg/g DW (water extraction). In stilbenes, three piceatannol hexosides were confirmed (Table 3). In the flavonols group, six kaempferol derivatives and seven quercetin derivatives were determined. Kaempferol pentosyl hexoside (1183.53 µg/g DW) and quercetin pentosyl hexoside 2 (1087.1 µg/g DW) were extremely high in the methanol extract (Table 3). In the methanol extract of A. pokeweed, the flavonols group was the most important group, since they alone accounted for 37% of the total analyzed phenolics (TAP), followed by hydroxycinnamic acids (22.8% TAP), stilbenes (22.1% TAP), flavanols (6.8% TAP), betalains (11.1% TAP) and flavones (0.05% TAP).

Table 4 shows the results of perennial ryegrass seed germination and the length of the sprouts in the control and A. pokeweed extracts. Similar to the case of J. knotweed, the water extract of A. pokeweed had a negative effect on seed germination. The germination of perennial ryegrass seeds treated with the extract of A. pokeweed was only 7.5% (extract concentration 1) and 15% (extract concentration 2), respectively, while in the control (treatment with water), as many as 97.5% of the perennial ryegrass seeds germinated. Treating the seeds with the aqueous extract of A. pokeweed (in both concentrations) had a characteristic negative effect on the growth of shoots and especially roots, because only the latter did not develop at all.

Treatment with an aqueous extract of A. pokeweed resulted in shoots 21.2 mm (conc. 2) to 21.5 mm (conc. 1) shorter than the average shoot length of the control (Table 4). The results showed that the aqueous extracts of both invasive species successfully reduced seed germination and also had an inhibitory effect on root and shoot growth of perennial ryegrass. A study by Kim et al. [24] found a similar effect of A. pokeweed. They found that a leaf extract of A. pokeweed inhibits the growth and development of Cassia mimosoides var. nomame. It has been reported that allelopathic compounds in plants may have a direct effect on metabolic processes and also on seed germination [8].

### 2.2. Effect of Solvent Type and Extraction Time on Phytochemical Extraction from Japanese Knotweed and American Pokeweed

We compared the efficiency of extraction of polyphenolic compounds with 80% methanol and 100% water. We chose 80% methanol because the literature [14,25,26] indicates that a combination of an organic solvent with water is usually the most effective solvent for the extraction of metabolites from plants, especially phenolic compounds. We used water as a second solvent because we were interested in whether we could achieve sufficiently good extraction of phenolics from plant material (J. knotweed, A. pokeweed) with the cheapest solvent that is also not harmful to the environment. With methanol solvent, we obtained a 14.5-fold higher content of flavonols, a 2.3-fold higher content of stilbenes, a 5-fold higher content of a quinone derivative and even a 16.4-fold higher content of flavones (apigenin hexoside) when extracting the J. knotweed (Table 1). That a water-alcohol mixture is more effective for the extraction of polyphenolics, especially representatives of the flavonols group, was also reported by Stanoeva et al. [27]. They found that the phenolic extraction yield was twice as high when 50% ethanol was used as compared to 100% water.

On the other hand, we obtained better extraction of hydroxycinnamic acids from J. knotweed with water, 1.5 times more than with 80% methanol (Table 3). The results of previous studies [28] also showed that water is a very good solvent for the extraction of phenolic acids. The same was demonstrated by Jimeez-Moreno et al. [26], who found the maximum extraction with 100% water in the case of gallic acid. This means that an aqueous solvent is suitable for phenolic substances with high polarity, including hydroxycinnamic acids.

Water extraction of American pokeweed gave a completely different picture than with a methanol extraction, since hydroxycinnamic acids took the top position, with a 48% share of TAP, followed by flavonols (16% TAP), stilbenes (19% TAP) and flavanols (17% TAP). As our results (Table 3) show, water is a very poor solvent for the extraction of stilbenes and flavonol glycosides from A. pokeweed. Al-Muwaly et al. [29] also stated that they achieved better extraction of phenolics from Iraqi sumac plant seeds with methanol than with ethanol and water, especially flavonoids. The results showed that methanol was a very effective solvent for the extraction of flavonoids and procyanidins from papaya leaves compared to water [11] and stilbenes from grape canes [15]. The reason that organic solvents such as methanol are more effective for the extraction of flavonoids is due to their polarity [30]. For less polar flavonoids, such as flavanones, isoflavones and flavonols, diethyl ether, hexane, acetone and chloroform can be used for better extraction efficiency [31]. However, it is interesting to note that the type of solvent in our study had no significant effect on the total content of hydroxycinnamic acids and flavanols in the extract of A. pokeweed.

The results of a comparison of the efficiency of the two solvents in the extraction of A. pokeweed showed that 80% methanol extracted 2.2 times more total polyphenolic substances, mainly due to the better extraction of flavonols (5.2 times more) and stilbenes (2.6 times more) (Table 3). Similar conclusions were reached by Muzolf-Panek [10], who found that a combination of a binary solvent (organic solvent and water) increased the efficiency of extraction of phenolic substances from plant material for various types of spices and herbs. The better solubility is partly related to the fact that water promotes diffusion of the extracted compounds throughout the plant tissue [32]. However, if one wants to achieve the maximum effect in extracting a wide range of different phenolic components, it must be considered that the water content in the binary solvent should not exceed 50%. The extraction efficiency of nonpolar substances will be significantly reduced [33]. From a safety point of view, water and ethanol are still the most suitable solvents for the extraction of secondary metabolites [10].

Some authors have previously studied the influence of temperature and incubation time on the extraction of total phenolic compounds [34,35,36]. However, these studies did not determine which individual phenolic compounds were extracted under the different experimental conditions, but generally measured only the total phenolic value determined spectrophotometrically. Although the effect of phenolic extraction is greater at a higher temperature, we wanted to determine the efficiency of extraction at a lower temperature (4 °C), because it is known that phenolic substances are oxidized and degraded at higher temperatures [12]. The information from our study could be very useful for choosing the optimal extraction method, depending on the further use of the obtained extract.

**Figure 1:**
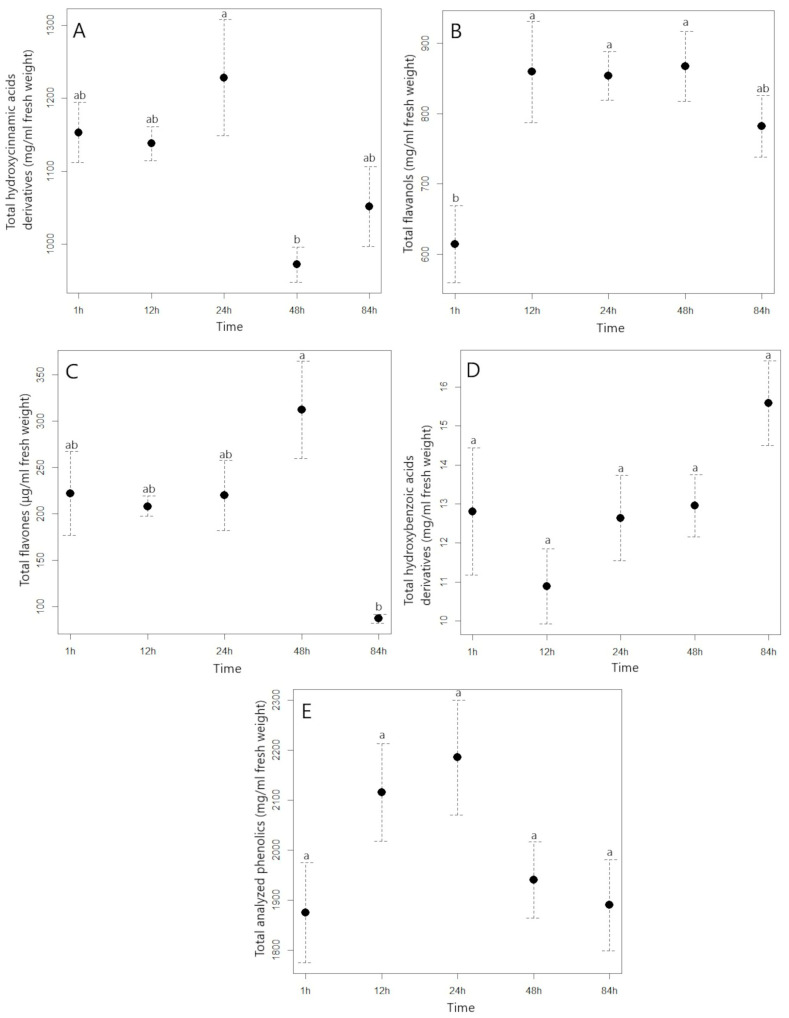
Average contents of total analyzed hydroxycinnamic acids (**A**), flavanols (**B**), flavones (**C**), hydroxybenzoic acid derivatives (**D**), and total analyzed phenolics (**E**) with standard error (mg/mL) in the aqueous extract of Japanese knotweed with different times of extraction. Different letters indicate statistically significant differences in the content of each phenolic group between different extraction times (T1–T5) (*p* ≤ 0.05). T1 represents a time of 1 h, T2 a time of 12 h, T3 a time of 24 h, T4 a time of 48 h, and T5 a time of 84 h.

**Figure 2:**
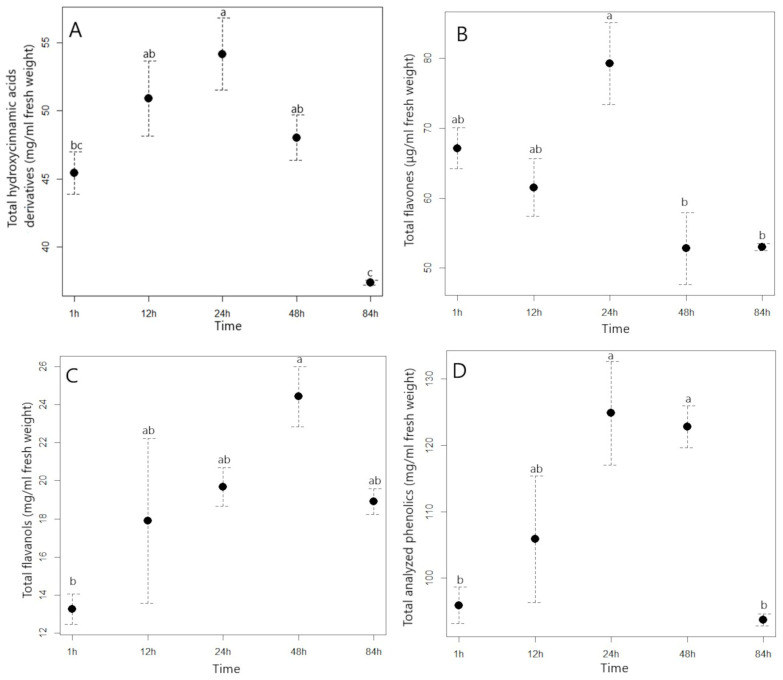
Average contents of total analyzed hydroxycinnamic acids (**A**), flavones (**B**), flavanols (**C**), and total analyzed phenolics (**D**) with standard error (mg/mL) in the aqueous extract of American pokeweed with different times of extraction. Different letters indicate statistically significant differences in the content of each phenolic group between different extraction times (T1–T5) (*p* ≤ 0.05). T1 represents a time of 1 h, T2 a time of 12 h, T3 a time of 24 h, T4 a time of 48 h, and T5 a time of 84 h.

In the preparation of water extracts from J. knotweed and A. pokeweed, the temperature was a constant (4 °C), but we were interested in how long it would take to reach the maximum content of extracted phenolic compounds. We found that the content of hydroxycinnamic acids (HCA) in the J. knotweed extract was fairly constant during the different extraction times (Figure 1A, Supplementary Table S3); only in the fourth term (48 h) was a decrease of 20% in the content observed. However, in the extract of A. pokeweed, the picture of the change in hydroxycinnamic acids was somewhat different (Figure 2A, Supplementary Table S4). Their content increased throughout the extraction time until the T3 (24 h) term (54.18 mg HCA/mL), when the HCA content in the extract was 19% higher than in the first term (1 h extraction, 45.42 mg HCA/mL). However, it is interesting to note that the HCA content in the extract of A. pokeweed decreased by 22% in the last term (T5—84 h) compared to the previous term (T4—48 h). In general, it can be recommended that for efficient extraction of HCA from plant material with an aqueous solvent and at low temperature (4 °C), an extraction time of 24 h should be used. Lovric et al. [37] also found that when blackthorn flowers were extracted at a temperature of 40–60 °C, increasing the extraction time beyond 5 min had no effect on an increase in the content of extracted hydroxycinnamic acids. Similarly, with thyme extraction at 50 °C, a better HCA extraction yield was not confirmed with a longer extraction time [36].

Examining the extraction efficiency of flavanols as a function of extraction time, it was observed that their content increased significantly in the second term (T2 12 h), i.e., by 40% compared with the first term (Figure 1B, Supplementary Table S2) and in the subsequent terms, the content of flavanols in the J. knotweed extract did not change significantly. A similar picture was found for the extract from A. pokeweed (Figure 2C, Supplementary Table S4), with which there was also a characteristic increase in flavanols in the second term (T2 12 h), after which their content remained fairly unchanged. It can be concluded from our results that 12 h is the appropriate time for optimal extraction of flavanols from J. knotweed and A. pokeweed with an aqueous solvent.

**Figure 3:**
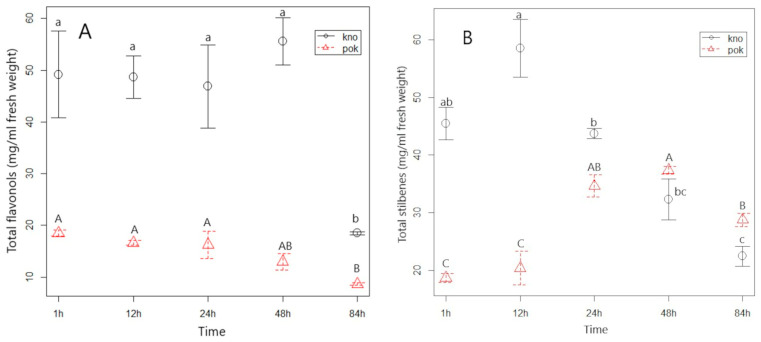
Average contents of total analyzed flavonols (**A**) and stilbenes (**B**) with standard error (mg/mL) in the aqueous extract of American pokeweed (pok) and Japanese knotweed (kno) with different times of extraction. Different small letters indicate statistically significant differences in the content of each phenolic group between different extraction times for Japanese knotweed (T1–T5) (*p* ≤ 0.05) and different capital letters indicate statistically significant differences in the content of each phenolic group between different extraction times for American pokeweed (T1–T5) (*p* ≤ 0.05). T1 represents a time of 1 h, T2 a time of 12 h, T3 a time of 24 h, T4 a time of 48 h, and T5 a time of 84 h.

The flavonol glycosides were extracted from J. knotweed and A. pokeweed quite rapidly, after only one hour. The content of flavonol glycosides in the aqueous extract remained fairly unchanged until term 4 (48 h after extraction) (Figure 3A, Supplementary Tables S3 and S4). Later, the content of total flavonols in the water extract of J. knotweed decreased by 66% and in the extract of A. pokeweed by 46%. This probably means that the flavonols had been degraded or it was due to adsorption/desorption processes. Park and Lee [38] reported that the lower polyphenolic content was due to a higher adsorption rate, which reduced the adsorbate yield. Ma et al. [39] also found that increasing the extraction time had a negative effect on the adsorption capacity of the rosavin component of goldenroot. It can therefore be concluded that a longer extraction time than 48 h is not suitable because of the instability of flavonols. A similar conclusion was reached by Vergara-Salinas et al. [36], who studied the efficiency of flavonols extraction from thyme. It was reported that a longer extraction time at a temperature of 50 °C did not lead to a higher flavonols extraction yield and that it even had a negative effect on their yield at a temperature of 150 °C [36].

The results from Figure 1C and Supplementary Table S3 show that with longer extraction time, the flavones content in the aqueous extract of J. knotweed increases up to 48 h after extraction, when we measured 313 ± 52.6 μg/mL, but at the end of the experiment we measured the lowest content, 86.7 ± 4.88 μg flavones/mL. In the extract of the A. pokeweed, the extraction time also had a significant effect on the flavones content (Figure 2B, Supplementary Table S4). After 24 h of extraction, the maximum flavones content (79.3 ± 5.89 μg/mL) was measured, and after 48 h of extraction, the flavone content was already 26.5 μg/mL lower than at term 3.

For stilbenes, extraction was most effective for J. knotweed in the first two terms (1 h to 12 h), while their content in the extract decreased significantly in the fourth and fifth terms, i.e., by 32% to 50% compared to the first term (1 h extraction) (Figure 3B, Supplementary Table S3). In the A. pokeweed extract, the maximum extraction efficiency of stilbenes from the plant material was recorded in the third (24 h) and fourth terms (48 h) (Figure 3B, Supplementary Table S4). In the case of stilbenes, it is difficult to give a general recommendation for their optimal time for successful extraction, but it can generally be said to be between 12 and 24 h. The type of plant material certainly has an influence on this. In an experiment conducted by Soural et al. [15], it was reported that the maximum concentration of stilbenes from grape cane was reached after four days of extraction, while their concentrations later decreased until the seventh day of extraction. In contrast, Romero-Perez et al. [40] recommended a shorter time for stilbenes extraction from grape skin: 30 min at a temperature of 60 °C. Certainly, the extraction efficiency depends mainly on the plant material to be extracted and the temperature. If the extraction of stilbenes is carried out at a lower temperature, a much longer extraction time must be used to obtain a sufficient stilbenes yield from the material [40].

Considering the tendency of extraction of the total analyzed compounds as a function of the extraction time, we found that their content in the J. knotweed extract was fairly constant (Figure 1E, Supplementary Table S3), while the maximum extraction of the total phenolics from A. pokeweed was achieved in the third (24 h) and fourth terms (48 h) (Figure 2D, Supplementary Table S4). The extraction efficiency of phenolics from plant material depends on many extraction factors: e.g., type of solvent, particle size of the material, extraction technique used (ultrasound-assisted extraction, microwave-assisted extraction, pressurized liquid extraction etc.), temperature, ratio of solid to liquid used, and extraction time. A longer extraction time means a higher efficiency of polyphenolic extraction until the plateau of the curve is reached. Spigno et al. [33] reported that the release of phenolic substances from grape marc increased at a temperature of 45 °C or 60 °C for up to 20 h, but thereafter the amount of extracted phenolics decreased.

## 3. Materials and Methods

### 3.1. Plant Material

We collected the aboveground parts of Japanese knotweed on 8 October 2020 near Ljubljana, 46°2′58 56″ N, 14°28′32 38″ E, and American pokeweed located at 46°3′28 71″ N, 14°27′54 49″ E. We collected the plants randomly from several locations. We selected plants that grew together and were at a similar stage of development. Shoots with leaves and inflorescences were collected from J. knotweed, shoots with leaves and fruits from A. pokeweed. After harvesting, the plant material was immediately taken to the laboratory.

### 3.2. Preparation of Aqueous Extracts

The aerial parts of the selected plants were finely chopped, water was poured over them, and they were well minced with a stick blender. Two concentrations of the aqueous extract were prepared: 1; a concentration of 1:0.2 g/mL (60 g of plant material plus 300 mL of water) and 2; a concentration of 2 for J. knotweed was 0.15 g/mL (30 g of J. knotweed plus 200 mL of water) and for A. pokeweed was 0.13 g/mL (40 g of A. pokeweed plus 300 mL of water). The prepared suspensions were shaken on a shaker at room temperature (20 °C) for one hour and then stored in a refrigerator overnight. After eleven hours, the water extracts were filtered through gauze and poured over the prepared seeds for the germination test.

### 3.3. Seed Germination

We chose a perennial ryegrass (*Lolium perenne* L.) as a model plant. Twenty seeds each were germinated on filter paper in Petri dishes (diameter 9 cm). We performed four replicates for each treatment (N = 80). Seeds were immediately irrigated with 5 mL of the aqueous extract from each invasive species. After two days, seeds were again irrigated with 4 mL of the extract. The seeds of the control treatment were irrigated with tap water. The Petri dishes were kept at room temperature and in daylight. After five days, we counted the germinated seeds and measured their shoot and root lengths. We considered seeds with at least 1 mm shoot or with at least 1 mm root to be successfully germinated seeds.

### 3.4. Effect of Solvent on the Extraction Efficiency of Phenolic Compounds

Two different solvents were used to extract phenolic substances from invasive plants. For optimal extraction of phenolics, we used 80% MeOH, with which extraction of phenolic substances is very successful according to the literature [41,42]. Two grams of plant material were weighed into numbered test tubes and covered with 8 mL of 80% methanol. All weighed samples of 8 mL of solvent used were accurately noted. We performed four replicates for each invasive species. The tubes were shaken thoroughly and then placed in an ice-cold ultrasonic water bath for 60 min. The test tubes were then stored in a refrigerator at 4 °C for 11 h. After twelve hours of extraction, the alcoholic extracts were centrifuged, and the supernatant filtered through PTFE filters into vials. The vials were then stored in a freezer at −20 °C until analysis.

Water was used as the second solvent. We used extracts that we had already prepared in the germination test, an extract with a concentration of 2:0.15 g/mL. We performed four replicates for each invasive plant. After twelve hours of extraction of the plant material, 4 mL of the suspension was filtered through gauze and then through cellulose filters into a vial. The vials were again stored in a freezer (−20 °C) until HPLC analysis.

### 3.5. Effect of Time on the Content of Phenolic Compounds in the Aqueous Extract

To compare the phenolic compound content as a function of extraction time, we used suspensions that we had already prepared in the experiment to inhibit seed germination with extracts of invasive species. We performed four replicates for each invasive species. We used water extracts of J. knotweed and A. pokeweed at a concentration of 2:0.15 g/mL. After shaking the suspensions on a shaker for 1 h (TERM 1 (T1) = extraction for 1 h), they were kept in a sealed bottle in a refrigerator for a specified time for further analysis: 11 h (T2 = 1 h + 11 h), 23 h (T3 = 1 h + 23 h), 47 h (T4 = 1 h + 47 h) and 83 h (T5 = 1 h + 83 h). The suspensions were first filtered through gauze and then through cellulose filters into a vial. The vials were stored in a freezer at −20 °C until analysis.

### 3.6. HPLC-DAD Analysis and Identification of Phenolic Compounds

Phenolic compounds were analyzed by HPLC (Dionex UltiMate 3000, Thermo Fisher Scientific, San Jose, CA, USA) with a DAD detector at a column temperature (Gemini C18, Phenomenex) of 25 °C. Compounds were detected at wavelengths of 280, 350 and 530 nm. Red pigments were analyzed at 530 nm. Two mobile phases were used for the separation of phenolic compounds: A; 0.1% formic acid/3% acetonitrile/96.9% double-distilled water and B; 0.1% formic acid/3% double-distilled water/96.9% acetonitrile, which were mixed according to the gradient method described by Mikulic-Petkovsek et al. [43]. The volume of injected samples was 20 μL and the flow rate of the mobile phase was set at 0.6 mL/min. The individual metabolites were identified by mass spectrometry (LTQ XL Linear Ion Trap Mass Spectrometer, Thermo Fisher Scientific, USA) with electrospray ionization (ESI) in negative and positive scanning under the modified parameters indicated by Mikulic-Petkovsek et al. [44]. The scanning range was from *m*/*z* 110 to 1700—data-dependent full scan. The electrospray ionization parameters were as follows: capillary temperature was 290 °C, sheet gas and auxiliary gas was 30 and 20 arb, respectively; ion spray voltage, 3.2 kV; capillary voltage, 24.0 V. Spectral data were elaborated using Excalibur software (Thermo Scientific). The phenolic compounds were confirmed based on the fragmentation products, comparison of the retention times of the corresponding standards, and comparison of the spectrum of individual peaks with the standards. The contents of phenolic compounds were calculated from the peak areas of the samples and with the corresponding standard curves of the phenolic compounds. The contents of phenolic compounds in invasive species were expressed in μg/g or mg/g DW (dry weight) and for water extracts in the experiment effect of extraction time in μg/mL or mg/mL FW (fresh weight).

### 3.7. Statistical Analysis

Data were statistically analyzed using the R 3.6.1 program with the R Commander interface. Statistically significant differences between two solvents were determined using the *t*-test at a 95% confidence level. The method of one-way analysis of variance (ANOVA) was used for statistical analysis of the germination test and the influence of extraction time on the content of secondary metabolites. For the characteristic traits ANOVA, multiple comparisons were performed with Tukey’s HSD test at a 95% confidence level. Means with standard errors and statistically significant differences between treatments were indicated by different letters.

## 4. Conclusions

Invasive alien plant species (IAPS) are a major problem nowadays because they have a negative impact on the environment, economy and human health. The use of IAPS has become an effective strategy to control them and can also help reduce the cost of their removal from the environment. Their usefulness depends on the alien plants species and their invasion. The results of comparing the efficiency of extraction of polyphenolic substances with two types of solvents (water and methanol) showed that we obtained better extraction efficiency of phenolic substances from both Japanese knotweed and A. pokeweed with methanol. A 1.3-fold higher content of total phenolics from J. knotweed and a 2.2-fold higher content from A. pokeweed were extracted with 80% methanol solvent than with water. Nevertheless, water remains the cheapest solvent for the extraction of constituents from plant parts that is safe for human health and the environment. The extraction time influenced the extraction efficiency of each phenolic compound. The maximum HCA content was extracted from A. pokeweed after 24 h. Extraction time did not significantly affect the extraction efficiency of HCA from J. knotweed. The maximum effect of extraction of flavanols was reached after 12 h and of flavonols after 1 h for both types of IAPS. However, the content of flavonols decreased significantly after 84 h of extraction. The results of the study have shown that water extracts of J. knotweed and A. pokeweed have an inhibitory effect on the germination of the seeds of perennial ryegrass. Aqueous extracts of both species also caused poorer root and shoot growth of perennial ryegrass. Additional studies should investigate other factors affecting the efficiency of phenolic extraction and the use of different concentrations of aqueous IAPS extracts to test the inhibition of germination and growth of some plants. The results of our study may be useful for various purposes in which individual IAPS could be beneficially used, e.g., in the agriculture, pharmaceuticals, wood and textile industries. Further studies are needed to verify whether the invasive species J. knotweed and A. pokeweed also have a phytotoxic effect on other species, especially weed species. If their phytotoxic effect is confirmed, these two IAPS could be used as natural weed control agents.

## Figures and Tables

**Table 1:** Contents of individual analyzed secondary metabolites with standard error (μg/g DW) in Japanese knotweed with two extraction solvents.

Phenolic Group	Methanol Extract	Water Extract
**Total hydroxybenzoic acids derivatives**	**0.00 ± 0.00**	**273.74 ± 24.17**
Gallic acid	/	16.367 ± 1366
Galloylhexoside 1	/	83.49 ± 7.64
Galloylhexoside 2	/	26.57 ± 3.41
**Total hydroxycinnamic acid derivatives**	**18,757.57 ± 2016.84 b**	**28,600.34 ± 592.94 a**
3-feruloylquinic acid	4.71 ± 0.55 a	8.28 ± 2.66 a
3-caffeoylquinic acid	751.44 ± 131.32 b	1752.37 ± 68.72 a
3-*p*-coumaroylquinic acid	73.19 ± 25.99 a	109.65 ± 5.82 a
4-caffeoylquinic acid	72.13 ± 7.49 b	110.05 ± 2.12 a
4-*p*-coumaroylquinic acid	28.55 ± 13.44 a	47.60 ± 7.06 a
5-*p*-coumaroylquinic acid 1	31.15 ± 7.38 a	18.62 ± 3.88 a
5-*p*-coumaroylquinic acid 2	71.04 ± 10.04 a	54.50 ± 6.21 a
5-caffeoylquinic acid 1	396.09 ± 61.43 a	414.68 ± 13.65 a
5-caffeoylquinic acid 2	73.68 ± 11.78 b	135.15 ± 5.14 a
Caftaric acid 1	16,805.52 ± 1744.96 b	25,640.47 ± 494.09 a
Caftaric acid 2	44.26 ± 7.08 b	81.19 ± 3.09 a
Caffeic acid hexoside	35.24 ± 11.91 a	31.53 ± 1.92 a
*cis*-coutaric acid	33.86 ± 8.02 a	20.24 ± 4.22 a
Dicaffeoylquinic acid 1	57.01 ± 1.74 a	3.86 ± 0.47 b
Dicaffeoylquinic acid 2	9.69 ± 1.98 a	1.54 ± 0.63 b
Ferulic acid pentoside	99.32 ± 14.04 a	76.20 ± 8.68 a
*trans*-coutaric acid	158.63 ± 15.72 a	84.45 ± 13.04 b
*p-*coumaric acid	2.71 ± 0.64 a	1.62 ± 0.34 a
*p*-coumaric acid hexoside	9.36 ± 3.16 a	8.37 ± 0.51 a
**Total flavanols**	**26,898.14 ± 2305.39 a**	**21,607.29 ± 1812.44 a**
Epicatechin	579.84 ± 92.73 b	1063.67 ± 40.43 a
Catechin hexoside	753.68 ± 307.75 a	552.90 ± 21.23 a
Catechin gallate	62.75 ± 3.47 a	18.54 ± 4.17 b
Procyanidin dimer 1	368.62 ± 105.33 b	831.28 ± 17.67 a
Procyanidin dimer 2	3966.48 ± 466.60 b	8588.65 ± 847.48 a
Procyanidin dimer 3	1380.08 ± 76.25 a	407.87 ± 91.61 b
Procyanidin tetramer 1	59.87 ± 9.57 b	109.82 ± 4.17 a
Procyanidin tetramer 2	5033.12 ± 496.56 a	1579.94 ± 110.01 b
Procyanidin tetramer 3	1157.61 ± 114.73 a	555.98 ± 61.67 b
Procyanidin tetramer 4	591.46 ± 32.68 a	174.80 ± 39.26 b
Procyanidin trimer 1	369.32 ± 57.28 a	386.66 ± 12.73 a
Procyanidin trimer 2	118.99 ± 14.00 b	257.66 ± 25.42 a
Procyanidin trimer 3	3390.53 ± 422.49 a	2297.43 ± 286.87 a
Procyanidin trimer 4	384.65 ± 54.38 a	295.10 ± 33.61 a
Procyanidin trimer 5	83.62 ± 11.82 a	64.15 ± 7.31 a
Procyanidin trimer 6	2841.72 ± 471.16 a	1929.60 ± 302.69 a
Procyanidin trimer 7	5755.80 ± 394.09 a	2493.25 ± 93.01 b
**Flavones**	**85.98 ± 2.63 a**	**5.23 ± 0.26 b**
Apigenin hexoside	85.98 ± 2.63 a	5.23 ± 0.26 b
**Total flavonols**	**17,630.81 ± 524.29 a**	**1223.46 ± 103.51 b**
Isorhamnetin hexoside	3.22 ± 0.20 a	0.21 ± 0.03 b
Kaempferol hexoside	40.17 ± 1.42 a	3.48 ± 0.39 b
Kaempferol-3-rhamnoside	165.97 ± 7.31 a	7.92 ± 1.11 b
Kaempferol-3-rutinoside	6.94 ± 0.28 a	0.41 ± 0.03 b
Quercetin acetyl hexoside	0.12 ± 0.02 a	0.03 ± 0.00 b
Quercetin dihexoside	89.52 ± 6.56 a	30.77 ± 1.00 b
Quercetin-3-arabinofuranoside	931.80 ± 30.98 a	10.77 ± 0.57 b
Quercetin-3-arabinopyranoside	245.01 ± 15.10 a	15.69 ± 1.98 b
Quercetin-3-galactoside	473.79 ± 20.21 a	46.56 ± 5.25 b
Quercetin-3-glucoside	517.85 ± 18.25 a	44.85 ± 4.97 b
Quercetin-3-rhamnoside	146,36.70 ± 447.69 a	951.59 ± 84.85 b
Quercetin-3-rutinoside	151.41 ± 6.68 a	85.70 ± 12.42 b
Quercetin-3-xyloside	346.18 ± 14.07 a	20.47 ± 1.76 b
Myricetin-3-rhamnoside	22.15 ± 1.91 a	5.02 ± 0.32 b
**Quinones**	**2.06 ± 0.42 a**	**0.41 ± 0.08 b**
Emodin hexoside	2.06 ± 0.42 a	0.41 ± 0.08 b
**Total stilbenes**	**3404.14 ± 307.54 a**	**1471.23 ± 126.74 b**
Astringin	13.70 ± 2.27 a	8.50 ± 0.99 a
*cis*-Resveratroloside	19.64 ± 0.65 a	0.24 ± 0.03 b
Piceatannol hexoside 1	345.69 ± 34.16 a	325.46 ± 49.51 a
Piceatannol hexoside 2	23.76 ± 1.31 a	8.16 ± 0.82 b
*trans*-Resveratroloside	1188.06 ± 65.64 a	407.98 ± 40.86 b
*trans*-Piceid 1	1628.03 ± 217.67 a	673.22 ± 92.44 b
*trans*-Piceid 2	185.25 ± 15.95 a	47.68 ± 4.17 b

Legend. Not analyzed /. Different letters in the rows indicate statistically significant differences in the content of individual phenolic substances between treatments (*p* ≤ 0.05).

**Table 2:** Average and standard error of germinated seed (%), length of shoot (mm) and roots (mm) of a perennial ryegrass (*Lolium perenne* L.) after treating with the water extracts of J. knotweed.

Plants	Concentration (g/mL)	Labels	Germination (%)	Shoot Length (mm)	Root Length (mm)
Control J. knotweed			97.50 ± 1.71 a	28.03 ± 1.09 a	20.57 ± 0.88 a
	0.2 0.15	1 2	50.00 ± 10.21 b 60.00 ± 7.91 b	13.38 ± 1.09 b 14.35 ± 0.96 b	1.03 ± 0.25 b 1.56 ± 0.30 b

Different letters in the columns indicate statistically significant differences of measured parameters between treatments (*p* ≤ 0.05).

**Table 3:** Contents of individual analyzed secondary metabolites with standard error (μg/g DW) in American pokeweed with two extraction solvents.

Compound	Methanol Extract	Water Extract
**Total betalains**	**1200.55 ± 170.07**	**0.00 ± 0.00**
Apiosylisobetanin	116.91 ± 9.70	/
Apiosylisobetanin	93.32 ± 25.75	/
Betanin isomer 1	97.19 ± 16.18	/
Betanin isomer 2	562.09 ± 47.72	/
Betanin isomer 3	331.04 ± 79.84	/
**Total hydroxycinamic acid derivatives**	**2471.39 ± 185.17 a**	**2355.75 ± 127.97 a**
3-*p*-coumaroylquinic acid	47.53 ± 3.52 a	45.88 ± 7.55 a
3-feruloylquinic acid	21.63 ± 1.45 a	19.22 ± 1.24 a
3-caffeoylquinic acid	452.80 ± 29.44 a	459.32 ± 44.51 a
4-caffeoylquinic acid	1456.87 ± 98.95 a	1248.71 ± 81.36 a
4-*p*-coumaroylquinic acid	34.65 ± 1.56 b	60.84 ± 7.32 a
5-caffeoylquinic acid 1	291.78 ± 101.72 a	322.34 ± 59.49 a
5-caffeoylquinic acid 2	42.28 ± 2.11 a	58.70 ± 10.04 a
5-*p*-coumaroylquinic acid 1	53.67 ± 3.39 a	69.34 ± 11.47 a
5-*p*-coumaroylquinic acid 2	11.82 ± 2.59 a	31.55 ± 8.35 a
Dicaffeoylquinic acid 1	11.16 ± 0.80 a	1.74 ± 0.06 b
Dicaffeoylquinic acid 2	32.62 ± 1.41 a	17.32 ± 1.50 b
*p*-coumaric acid hexoside	12.99 ± 5.24 a	18.69 ± 5.28 a
*p-*coumaric acid	1.61 ± 0.10 a	2.10 ± 0.35 a
**Total flavanols**	**735.67 ± 109.60 a**	**827.82 ± 199.75 a**
Epicatechin	139.24 ± 6.96 a	193.33 ± 33.06 a
Catechin	62.83 ± 4.65 a	60.66 ± 9.99 a
Catechin gallate	213.44 ± 41.40 a	229.53 ± 62.86 a
Catechin hexoside	320.16 ± 62.10 a	344.30 ± 94.29 a
**Flavone**	**5.17 ± 0.22 a**	**2.85 ± 0.19 b**
Apigenin dihexoside	5.17 ± 0.22 a	2.85 ± 0.19 b
**Total flavonols**	**4045.24 ± 168.42 a**	**770.05 ± 21.32 b**
Kaempferol glucuronyl dihexoside	65.02 ± 3.30 a	9.70 ± 1.19 b
Kaempferol glucuronyl pentoside hexoside	60.72 ± 4.04 a	4.29 ± 1.07 b
Kaempferol hexoside	272.05 ± 19.39 a	42.51 ± 1.57 b
Kaempferol-3-rutinoside	291.74 ± 4.97 a	80.32 ± 1.59 b
Kaempferol pentosyl hexoside	1183.53 ± 39.52 a	122.43 ± 1.55 b
Kaempferol rhamnosyl dihexoside	91.50 ± 4.38 a	37.66 ± 3.00 b
Quercetin-3-xyloside	22.62 ± 1.35 b	32.98 ± 3.30 a
Quercetin-3-arabinofuranoside	4.08 ± 0.29 a	0.64 ± 0.02 b
Quercetin dihexoside	36.69 ± 3.95 a	13.41 ± 3.17 b
Quercetin pentosyl hexoside 1	184.51 ± 9.12 a	39.03 ± 1.75 b
Quercetin pentosyl hexoside 2	1087.10 ± 52.53 a	193.92 ± 4.11 b
Quercetin rhamnosyl hexoside	134.70 ± 6.66 a	28.49 ± 1.28 b
Quercetin-3-glucoside	611.00 ± 35.69 a	164.68 ± 2.91 b

Legend. Not analyzed /. Different letters in the rows indicate statistically significant differences in the content of individual phenolic substances between treatments (*p* ≤ 0.05).

**Table 4:** Average and standard error of germinated seed (%), length of shoot (mm) and roots (mm) of a perennial ryegrass (*Lolium perenne* L.) after treating with the water extracts of American pokeweed.

Plants	Concentration (g/mL)	Labels	Germination (%)	Shoot Length (mm)	Root Length (mm)
Control			97.50 ± 1.71 a	28.03 ± 1.09 a	20.57 ± 0.88 a
A. pokeweed	0.2 0.13	1 2	7.50 ± 1.44 b 15.00 ± 5.77 b	6.50 ± 1.61 b 6.83 ± 0.91 b	0.00 ± 0.00 b 0.00 ± 0.00 b

Different letters in the columns indicate statistically significant differences of measured parameters between treatments (*p* ≤ 0.05).

## Data Availability

The data presented in this study are available in the article and the Supplementary Material.

## Supplementary Materials

The following supporting information can be downloaded at: https://www.mdpi.com/article/10.3390/plants11223053/s1, Table S1: Identification of phenolic compounds in Japanese knotweed in negative ionization with HPLC-MS and MS^2^/MS^3^. Table S2: Identification of phenolic compounds in American pokeweed in negative ionization with HPLC-MS and MS^2^/MS^3^. Table S3: Content of phenolic compounds and standard error (mg/mL) (quinones and flavones are expressed in μg/mL) in Japanese knotweed water extracts with different times of extraction. Different letters in rows denote statistically significant differences between different times with Tukey’s HSD test. (T1 = 1 h of extraction, T2 = 12 h of extraction, T3 = 24 h of extraction, T4 = 48 h of extraction and T5 = 84 h of extraction). Table S4: Content of phenolic compounds and standard error (mg/mL) (flavones are expressed in μg/mL) in American pokeweed water extracts with different times of extraction. Different letters in rows denote statistically significant differences between different times with Tukey’s HSD test. (T1 = 1 h of extraction, T2 = 12 h of extraction, T3 = 24 h of extraction, T4 = 48 h of extraction and T5 = 84 h of extraction).
