# Supplementary material for: HPLC-DAD-MS Identification and Quantification of Phenolic Components in Japanese Knotweed and American Pokeweed Extracts and Their Phytotoxic Effect on Seed Germination
Source: Plants (Basel). 2022 Nov 11;11(22):3053. doi: 10.3390/plants11223053 (PMC9693380; doi:10.3390/plants11223053)
Supplement: Supplementary file 1 [file plants-11-03053-s001.zip › Supplementary table S1.pdf]

Supplementary Table S1. Identification of phenolic compounds in Japanese knotweed in negative ionization with HPLC-MS and MS^2/MS^3

Phenolic group	λ (nm)	$[M-H]^-$ (*m/z*)	MS^2 (*m/z*)	MS^3 (*m/z*)
**Hydroxybenzoic acids derivatives**				
Gallic acid	271	169	125	
Galloylhexoside 1	276	331	169	
Galloylhexoside 2	276	331	169	
**Hydroxycinnamic acid derivatives**				
3-Feruloylquinic acid	320	367	193,134	149
3-Caffeoylquinic acid	234, 326	353	191, 179, 135	173, 127, 85
3-*p*-Coumaroylquinic acid	312	337	163	
4-Caffeoylquinic acid	234, 328	353	173, 179	
4-*p*-Coumaroylquinic acid	312	337	173, 163, 155, 137, 191	
5-*p*-Coumaroylquinic acid 1	312	337	191,173,163	
5-*p*-Coumaroylquinic acid 2	311	337	191,163,173	
5-Caffeoylquinic acid 1	234,328	353	191,179,135	173,127,85
5-Caffeoylquinic acid 2	234,328	353	191,179,135	
cis-Coutaric acid	310	295	163	
Dicaffeoylquinic acid 1	334	515	353	191
Dicaffeoylquinic acid 2	333	515	353	191
Caffeic acid hexoside	330	341	179	
p-Coumaric acid hexoside	311	325	163	
Caftaric acid 1	328	311	149,179	
Caftaric acid 2	328	311	149,179	
Ferulic acid pentoside	290,310	325	163	
trans-Coutaric acid	310	295	163	
p-Coumaric acid	310	163	119	
**Flavanols**				
Epicatechin	234,279	289	245	
Catechin hexoside	235,280	451	289	
Catechin gallate	235,280	441	289	
Procyanidin dimer 1	278	577	425,451,407,289	
Procyanidin dimer 2	278	577	425,451,407,289	
Procyanidin dimer 3	279	577	425,407,289	
Procyanidin tetramer 1	234,278	1153	1135, 1027, 983, 865, 863, 577	
Procyanidin tetramer 2	234,278	1153	1135, 1027, 983, 865, 863, 695,577,	
Procyanidin tetramer 3	234,278	1153	1027, 983, 865, 577, 575	
Procyanidin tetramer 4	234,278	1153	1135, 1027, 983, 865, 863, 577	
Procyanidin trimer 1	234,278	865	577,407,289	
Procyanidin trimer 2	234,278	865	577,425,407,289	
Procyanidin trimer 3	235,280	865	577,425,407,289	
Procyanidin trimer 4	236,279	865	577,425,289	
Procyanidin trimer 5	234,280	865	577,407,289	
Procyanidin trimer 6	235,279	865	577,425,407,289	
Procyanidin trimer 7	234,280	865	577,425,407,289	
**Flavones**				
Apigenin hexoside	268,334	431	269	
**Flavonols**				
Isorhamnetin hexoside	255,352	477	315	
Kaempferol hexoside	266,346	447	285	
Kaempferol-3-rhamnoside	266, 347	431	285	
Kaempferol-3-rutinoside	264,345	593	285	
Quercetin-3-xyloside	356,255	433	301	
Quercetin-3-arabinofuranoside	355,255	433	301	
Quercetin acetyl hexoside	255,355	505	463	301
Quercetin dihexoside	255,356	625	301	
Quercetin-3-arabinopyranoside	256, 352	433	301	
Quercetin-3-galactoside	256,356	463	301	
Quercetin-3-glucoside	255,355	463	301	

**Phenolic group**	**λ (nm)**	**[M-H]⁻ (*m/z*)**	**MS² (*m/z*)**	**MS³ (*m/z*)**
Quercetin-3-rhamnoside	266,356	447	301	
Quercetin-3-rutinoside	255,355	609	301	
Myricetin-3-rhamnoside	261, 349	463	317	
**Quinones**				
Emodin hexoside	282,450	431	269	
**Stilbenes**				
Astringin	331,305	405	243,225,199	
cis-Resveratroloside	311	389	227,371,209	
Piceatannol hexoside 1	324,290,306	405	243	
Piceatannol hexoside 2	324,290,306	405	243	
trans-Resveratroloside	311	389	227	
trans-Piceid 1	318,306,229	389	227,185	
trans-Piceid 2	318,306,229	389	227,185	
