# Supplementary material for: HPLC-DAD-MS Identification and Quantification of Phenolic Components in Japanese Knotweed and American Pokeweed Extracts and Their Phytotoxic Effect on Seed Germination
Source: Plants (Basel). 2022 Nov 11;11(22):3053. doi: 10.3390/plants11223053 (PMC9693380; doi:10.3390/plants11223053)
Supplement: Supplementary file 1 [file plants-11-03053-s001.zip › Supplementary table S2.pdf]

Supplementary Table S2 Identification of phenolic compounds in American pokeweed in negative ionization with HPLC-MS and MS^2/MS^3

Phenolic group	λ (nm)	$[M-H]^-$ (*m/z*)	MS^2 (*m/z*)	MS^3 (*m/z*)
**Hydroxycinamic acid derivatives**				
3-*p*-Coumaroylquinic acid	312	337	163	
3-Feruloylquinic acid	320	367	193,134	149
3-Caffeoylquinic acid	234, 326	353	191, 179, 135	173, 127, 85
4-Caffeoylquinic acid	234, 328	353	173, 179	
4-*p*-Coumaroylquinic acid	312	337	173, 163, 155, 137, 191	
5-Caffeoylquinic acid 1	234,328	353	191,179,135	173,127,85
5-Caffeoylquinic acid 2	234,328	353	191,179,135	
5-*p*-Coumaroylquinic acid 1	312	337	191,173,163	
5-*p*-Coumaroylquinic acid 2	311	337	191,163,173	
Dicaffeoylquinic acid 1	246,316	515	353	179,173
Dicaffeoylquinic acid 2	246, 316	515	353	179,173
p-Coumaric acid hexoside	322,275	325	163,119	
p-Coumaric acid	310	163	119	
**Flavanols**				
Epicatechin	234,279	289	245	
Catechin	234,279	289	245	
Catechin gallate	235,280	441	289,169,135	
Catechin hexoside	235,280	451	289	
**Flavones**				
Apigenin dihexoside	268,331	431	269	
**Flavonols**				
Kaempferol glucuronyl dihexoside	348, 265	785	609	285
Kaempferol glucuronyl pentoside hexoside	347, 266	755	579	285
Kaempferol hexoside	266,346	447	285	
Kaempferol-3-rutinoside	264,345	593	285	
Kaempferol pentosyl hexoside	266, 347	579	447	285
Kaempferol rhamnosyl dihexoside	265, 346	755	609	285
Quercetin-3-xyloside	356,255	433	301	
Quercetin-3-arabinofuranoside	355,255	433	301	
Quercetin dihexoside	255,356	625	301	
Quercetin pentosyl hexoside 1	256,355	595	463	301
Quercetin pentosyl hexoside 2	256,356	595	463	301
Quercetin rhamnosyl hexoside	255,355	609	463	301
Quercetin-3-glucoside	255,355	463	301	
**Stilbenes**				
Piceatannol hexoside 1	324	405	243	
Piceatannol hexoside 2	324	405	243	
Piceatannol hexoside 3	324	405	243	
