# Supplementary material for: HPLC-DAD-MS Identification and Quantification of Phenolic Components in Japanese Knotweed and American Pokeweed Extracts and Their Phytotoxic Effect on Seed Germination
Source: Plants (Basel). 2022 Nov 11;11(22):3053. doi: 10.3390/plants11223053 (PMC9693380; doi:10.3390/plants11223053)
Supplement: Supplementary file 1 [file plants-11-03053-s001.zip › Supplementary table S3.pdf]

Supplementary Table S3. Content of phenolic compounds and standard error (mg/ml) (quinones and flavones are expressed in µg/ml) in Japanese knotweed water extracts with different times of extraction. Different letters in rows denote statistically significant differences between different times with Tukey's HSD test. (T1 = 1 hour of extraction, T2 = 12 hours of extraction, T3 = 24 hours of extraction, T4 = 48 hours of extraction and T5 = 84 hours of extraction)

Phenolic group	T1	T2	T3	T4	T5
**Total hydroxybenzoic acids**	**12.81 ± 1.63 a**	**10.89 ± 0.96 a**	**12.63 ± 1.08 a**	**12.95 ± 0.80 a**	**15.59 ± 1.09 a**
Gallic acid	7.37 ± 0.99 a	6.51 ± 0.54 a	7.55 ± 0.61 a	7.76 ± 0.41 a	9.69 ± 0.74 a
Galloylhexoside 1	3.81 ± 0.60 a	3.32 ± 0.30 a	3.64 ± 0.38 a	3.92 ± 0.30 a	4.18 ± 0.24 a
Galloylhexoside 2	1.63 ± 0.13 ab	1.06 ± 0.14 b	1.45 ± 0.18 ab	1.27 ± 0.11 ab	1.72 ± 0.13 a
**Total hydroxycinnamic acid**	**1153.15 ± 41.11**	**1137.81 ± 23.59**	**1228.37 ± 79.87**	**971.83 ± 24.09**	**1051.61 ± 54.79**
3-Feruloylquinic acid	0.19 ± 0.07 b	0.33 ± 0.11 ab	0.43 ± 0.02 ab	0.49 ± 0.01 a	0.50 ± 0.02 a
3-Caffeoylquinic acid	68.60 ± 2.16 a	69.72 ± 2.73 a	77.69 ± 3.47 a	69.39 ± 2.04 a	70.58 ± 2.49 a
3-*p*-Coumaroylquinic acid	3.76 ± 0.24 a	4.36 ± 0.23 a	4.37 ± 0.14 a	3.84 ± 0.06 a	3.85 ± 0.12 a
4-Caffeoylquinic acid	4.47 ± 0.16 ab	4.38 ± 0.08 ab	4.71 ± 0.32 a	3.67 ± 0.09 b	4.02 ± 0.22 ab
4-*p*-Coumaroylquinic acid	1.71 ± 0.27 b	1.89 ± 0.28 ab	2.53 ± 0.06 ab	2.79 ± 0.17 a	1.96 ± 0.33 ab
5-*p*-Coumaroylquinic acid 1	16.94 ± 1.30 a	16.50 ± 0.54 a	19.35 ± 1.09 a	16.28 ± 0.48 a	16.41 ± 0.60 a
5-*p*-Coumaroylquinic acid 2	3.48 ± 0.11 b	5.38 ± 0.20 a	5.20 ± 0.47 a	5.31 ± 0.15 a	5.63 ± 0.12 a
5-Caffeoylquinic acid 1	0.72 ± 0.11 a	0.74 ± 0.15 a	0.90 ± 0.02 a	0.68 ± 0.05 a	0.64 ± 0.12 a
5-Caffeoylquinic acid 2	1.76 ± 0.34 a	2.17 ± 0.25 a	2.18 ± 0.05 a	1.83 ± 0.17 a	1.41 ± 0.27 a
cis-Coutaric acid	0.78 ± 0.12 a	0.81 ± 0.17 a	0.98 ± 0.03 a	0.74 ± 0.05 a	0.70 ± 0.13 a
Dicaffeoylquinic acid 1	0.13 ± 0.04 ab	0.15 ± 0.02 ab	0.13 ± 0.03 ab	0.21 ± 0.04 a	0.07 ± 0.01 b
Dicaffeoylquinic acid 2	0.02 ± 0.01 a	0.06 ± 0.03 a	0.08 ± 0.03 a	0.06 ± 0.04 a	0.05 ± 0.05 a
Caftaric acid hexoside	1.11 ± 0.20 a	1.25 ± 0.08 a	1.38 ± 0.08 a	1.16 ± 0.12 a	0.45 ± 0.09 b
p-Coumaric acid hexoside	0.29 ± 0.05 a	0.33 ± 0.02 a	0.37 ± 0.02 a	0.31 ± 0.03 a	0.12 ± 0.02 b
Caftaric acid 1	1042.10 ± 36.29	1020.06 ± 19.66	1098.53 ± 74.25	855.74 ± 20.02	937.45 ± 50.82
Caftaric acid 2	2.09 ± 0.07 b	3.23 ± 0.13 a	3.12 ± 0.29 a	3.19 ± 0.09 a	3.38 ± 0.07 a
Ferulic acid pentoside	2.47 ± 0.48 a	3.03 ± 0.35 a	3.05 ± 0.07 a	2.56 ± 0.24 a	1.97 ± 0.38 a
trans-Coutaric acid	2.47 ± 0.34 a	3.36 ± 0.52 a	3.30 ± 0.25 a	3.51 ± 0.64 a	2.35 ± 0.50 a
p-Coumaric acid	0.06 ± 0.01 a	0.06 ± 0.01 a	0.08 ± 0.00 a	0.06 ± 0.00 a	0.06 ± 0.01 a
**Total flavanols**	**614.34 ± 54.94**	**859.60 ± 72.10**	**853.90 ± 34.44**	**867.67 ± 49.64**	**781.91 ± 43.77**
Epicatechin	27.41 ± 0.89 b	42.32 ± 1.61 a	40.92 ± 3.73 a	41.82 ± 1.20 a	44.28 ± 0.92 a
Catechin hexoside	25.10 ± 1.98 a	22.00 ± 0.85 a	21.03 ± 0.89 a	15.92 ± 0.80 b	13.78 ± 0.53 b
Catechin gallate	0.76 ± 0.12 a	0.74 ± 0.17 a	0.56 ± 0.14 a	0.57 ± 0.09 a	0.27 ± 0.07 a
Procyanidin dimer 1	30.51 ± 1.55 bc	33.07 ± 0.70 ab	37.05 ± 0.58 a	31.35 ± 1.31 bc	26.70 ± 1.30 c
Procyanidin dimer 2	194.39 ± 39.36	341.68 ± 33.72	361.96 ± 14.31	415.67 ± 9.94 a	418.32 ± 12.91
Procyanidin dimer 3	16.75 ± 2.69 a	16.23 ± 3.65 a	12.35 ± 3.06 a	12.48 ± 2.00 a	5.95 ± 1.59 a
Procyanidin tetramer 1	2.83 ± 0.09 b	4.37 ± 0.17 a	4.23 ± 0.39 a	4.32 ± 0.12 a	4.57 ± 0.10 a
Procyanidin tetramer 2	55.31 ± 5.80 a	62.86 ± 4.38 a	69.73 ± 6.59 a	58.85 ± 3.09 a	56.57 ± 5.29 a
Procyanidin tetramer 3	15.63 ± 0.71 a	22.12 ± 2.45 a	24.08 ± 1.80 a	23.99 ± 3.06 a	20.34 ± 2.59 a
Procyanidin tetramer 4	7.18 ± 1.15 a	6.95 ± 1.56 a	5.29 ± 1.31 a	5.35 ± 0.84 a	2.55 ± 0.68 a
Procyanidin trimer 1	15.80 ± 1.21 a	15.38 ± 0.51 a	18.04 ± 1.02 a	15.18 ± 0.45 a	15.31 ± 0.56 a
Procyanidin trimer 2	5.83 ± 1.18 b	10.25 ± 1.01 a	10.86 ± 0.43 a	12.47 ± 0.30 a	12.55 ± 0.39 a
Procyanidin trimer 3	79.60 ± 12.98	91.40 ± 11.41 a	84.95 ± 8.41 ab	85.15 ± 9.72 ab	46.19 ± 4.10 b
Procyanidin trimer 4	9.55 ± 1.86 a	11.74 ± 1.34 a	11.82 ± 0.27 a	9.92 ± 0.94 a	7.63 ± 1.45 a
Procyanidin trimer 5	2.08 ± 0.40 a	2.55 ± 0.29 a	2.57 ± 0.06 a	2.16 ± 0.20 a	1.66 ± 0.32 a
Procyanidin trimer 6	59.48 ± 9.07 a	76.77 ± 12.04 a	70.47 ± 4.25 a	73.34 ± 8.08 a	55.23 ± 11.99 a
Procyanidin trimer 7	66.13 ± 9.03 ab	99.19 ± 3.70 a	78.00 ± 6.64 ab	59.16 ± 10.14 b	50.02 ± 11.72 b
**Total flavones**	**222.01 ± 45.25**	**207.98 ±**	**219.90 ± 37.95**	**312.58 ± 52.56**	**86.74 ± 4.88 b**
Apigenin hexoside	222.01 ± 45.25	207.98 ± 10.45	219.90 ± 37.95	312.58 ± 52.56	86.74 ± 4.88 b
**Total flavonols**	**49.17 ± 8.41 a**	**48.67 ± 4.12 a**	**46.90 ± 8.04 a**	**55.60 ± 4.59 a**	**18.48 ± 0.30 b**
Isorhamnetin hexoside	0.005 ± 0.001b	0.008 ± 0.001	0.01 ± 0.001 a	0.006 ± 0.001 b	0.002 ± 0.001 c
Kaempferol hexoside	0.14 ± 0.03 a	0.14 ± 0.02 a	0.12 ± 0.02 ab	0.12 ± 0.01 ab	0.05 ± 0.001 b
Kaempferol-3-rhamnoside	0.25 ± 0.05 ab	0.32 ± 0.04 ab	0.25 ± 0.06 ab	0.37 ± 0.08 a	0.09 ± 0.005 b
Kaempferol-3-rutinoside	0.007 ± 0.001	0.02 ± 0.001 a	0.01 ± 0.001 bc	0.01 ± 0.001 b	0.005 ± 0.0003
Quercetin-3-xyloside	0.42 ± 0.06 b	0.82 ± 0.07 a	0.50 ± 0.08 ab	0.44 ± 0.14 b	0.24± 0.03 b
Quercetin-3-arabinofuranoside	0.45 ± 0.69 ab	428.48 ± 0.02	0.43 ± 0.10 ab	0.89 ± 0.21 a	0.16 ± 0.06 b
Quercetin acetyl hexoside	0.0005 ±	0.001 ± 0.0002	0.0006 ± 0.0003	0.001 ± 0.0004	0.001 ± 0.0004

Phenolic group	T1	T2	T3	T4	T5
Quercetin dihexoside	1.01 ± 0.14 b	1.22 ± 0.04 b	2.24 ± 0.36 ab	1.51 ± 0.09 a	1.07 ± 0.08 b
Quercetin-3-arabinopyranoside	0.43 ± 0.08 b	0.62 ± 0.08 ab	0.80 ± 0.04 a	0.46 ± 0.09 b	0.11 ± 0.04 c
Quercetin-3-galactoside	1.79 ± 0.40 a	1.85 ± 0.21 a	1.45 ± 0.32 ab	1.64 ± 0.17 ab	0.62 ± 0.06 b
Quercetin-3-glucoside	1.80 ± 0.36 a	1.78 ± 0.2 a	1.58 ± 0.31 ab	1.49 ± 0.16 ab	0.58 ± 0.04 b
Quercetin-3-rhamnoside	40.18 ± 7.11 a	37.86 ± 3.38 a	36.08 ± 6.77 a	45.96 ± 3.70 a	13.89 ± 0.51 b
Quercetin-3-rutinoside	2.60 ± 0.39 ab	3.41 ± 0.49 a	3.19 ± 0.46 ab	2.56 ± 0.26 ab	1.58 ± 0.15 b
Myricetin-3-rhamnoside	0.09 ± 0.007 c	0.20 ± 0.01 ab	0.24 ± 0.02 a	0.15 ± 0.02 bc	0.08 ± 0.02 c
**Quinones**	**59,08 ± 7,70 a**	**107,39 ± 22,02**	**109,09 ± 34,88**	**105,73 ± 43,24**	**58,86 ± 11,72 a**
Emodin hexoside	59.08 ± 7.70 a	107.39 ± 22.02	109.09 ± 34.88	105.73 ± 43.24	58.86 ± 11.72 a
**Total stilbenes**	**45.46 ± 2.80 ab**	**58.53 ± 5.04 a**	**43.73 ± 0.90 b**	**32.27 ± 3.57 bc**	**22.43 ± 1.750 c**
Astringin	0.26 ± 0.02 a	0.34 ± 0.04 a	0.34 ± 0.02 a	0.34 ± 0.02 a	0.31 ± 0.04 a
cis-Resveratroloside	0.01 ± 0.001 ab	0.01 ± 0.001 ab	0.01 ± 0.003 ab	0.02 ± 0.01 a	0.002 ± 0.0002
Piceatannol hexoside 1	16.46 ± 1.05 a	12.95 ± 1.97 ab	17.87 ± 0.88 a	10.20 ± 0.73 b	9.53 ± 0.65 b
Piceatannol hexoside 2	0.24 ± 0.01 ab	0.33 ± 0.03 a	0.18 ± 0.03 bc	0.18 ± 0.01 bc	0.13 ± 0.01 c
trans-Resveratroloside	12.16 ± 0.66 ab	16.23 ± 1.63 a	8.94 ± 1.49 bc	9.05 ± 0.32 bc	6.25 ± 0.27 c
trans-Piceid 1	15.54 ± 1.26 b	26.78 ± 3.68 a	14.36 ± 1.19 bc	11.27 ± 2.43 bc	5.56 ± 1.91 c
trans-Piceid 2	0.80 ± 0.07 b	1.90 ± 0.17 a	2.03 ± 0.18 a	1.21 ± 0.19 b	0.65 ± 0.14 b
**Total polyphenolics**	**1875.20 ± 99.89**	**2115.82 ± 97.55**	**2185.86 ±**	**1940.74 ± 75.74**	**1890.16 ± 90.72**
