# Supplementary material for: HPLC-DAD-MS Identification and Quantification of Phenolic Components in Japanese Knotweed and American Pokeweed Extracts and Their Phytotoxic Effect on Seed Germination
Source: Plants (Basel). 2022 Nov 11;11(22):3053. doi: 10.3390/plants11223053 (PMC9693380; doi:10.3390/plants11223053)
Supplement: Supplementary file 1 [file plants-11-03053-s001.zip › Supplementary table S4.pdf]

Supplementary Table S4. Content of phenolic compounds and standard error (mg/ml) (flavones are expressed in μg/ml) in American pokeweed water extracts with different times of extraction. Different letters in rows denote statistically significant differences between different times with Tukey's HSD test. (T1 = 1 hour of extraction, T2 = 12 hours of extraction, T3 = 24 hours of extraction, T4 = 48 hours of extraction and T5 = 84 hours of extraction)

Phenolic group	T1	T2	T3	T4	T5
**Hydroxycinamic acid derivatives**	**45.42 ±1.56 bc**	**50.91 ± 2.77 ab**	**54.18 ± 2.66 a**	**48.03 ± 1.67 ab**	**37.36 ± 0.17 c**
3-*p*-Coumaroylquinic acid	0.74 ± 0.06 c	0.99 ± 0.16 bc	1.32 ± 0.06 ab	1.48 ± 0.06 a	1.02 ± 0.06 bc
3-Feruloylquinic acid	0.42 ± 0.01 a	0.42 ± 0.03a	0.38 ± 0.02 a	0.36 ± 0.002 ab	0.31 ± 0.007 b
3-Caffeoylquinic acid	9.72 ± 0.28 a	9.93 ±0.96 a	9. 11 ± 0.24 ab	7.90 ± 0.42 ab	7.01 ± 0.23 b
4-Caffeoylquinic acid	27.79 ± 0.98 a	26.99 ± 1.76 ab	24.14 ± 0.96 ab	22.79 ± 0.14 bc	18.86 ±0. 48 c
4-*p*-Coumaroylquinic acid	1.08 ± 0.07 b	1.32 ± 0.16 b	1.71 ± 0.07 a	1.72 ± 0.03 a	0.96 ± 0.02 b
5-Caffeoylquinic acid 1	2.40 ± 0.3 c	7.00 ± 1.29 b	10.98 ± 0.87 a	7.34± 0.95 ab	3.98 ± 0.35 bc
5-Caffeoylquinic acid 2	1.26 ± 0.12 b	1.27 ± 0.22 b	2.46 ± 0.15 a	2.42 ± 0.07 a	2.19 ± 0.03 a
5-*p*-Coumaroylquinic acid 1	1.05 ± 0.14 b	1.50 ± 0.25 ab	1.98 ± 0.08 a	1.93 ± 0.08 a	1.43 ± 0.05 ab
5-*p*-Coumaroylquinic acid 2	0.20 ± 0.12 b	0.68 ± 0.18 ab	1.12 ± 0.22 a	0.87 ± 0.06 a	0.59 ± 0.02 ab
Dicaffeoylquinic acid 1	0.04 ± 0.002 a	0.04 ± 0.001 a	0.04 ± 0.01 a	0.03 ± 0.007 a	24.27 ± 1.31 a
Dicaffeoylquinic acid 2	0.42 ± 0.02 a	0.37 ± 0.03 a	0.40 ± 0.11 a	0.31 ± 0.06 a	334.86 ± 3.14 a
p-Coumaric acid hexoside	0.26 ± 0.07 c	0.40 ± 0.11 bc	0.47 ± 0.03 bc	0.82 ± 0.09 a	0.61 ± 0.06 ab
p-Coumaric acid	0.03 ± 0.004 b	0.05 ± 0.01 ab	0.06 ± 0.01 a	0.06 ± 0.002 a	0.04 ± 0.002 ab
**Flavanols**	**13.27 ± 0.80 b**	**17.89 ± 4.32 ab**	**19.68 ± 1.00 ab**	**24.4 ± 1.58 a**	**18.90 ± 0.67 ab**
Epicatechin	4.14 ± 0.4 b	4.18 ± 0.72 b	8.11 ± 0.50 a	7.97 ± 0.24 a	7.21 ± 0.10 a
Catechin	0.98 ± 0.08 c	1. 31 ± 0.22 bc	1.75 ± 0.08 ab	1.96 ± 0.07 a	1.34 ± 0.08 bc
Catechin gallate	3.26 ± 0.14 a	4.96 ± 1.26 a	3.93 ± 0.19 a	5.80 ± 0.51 a	4.14 ± 0.26 a
Catechin hexoside	4.89 ± 0.21 a	7.44 ± 2.04 a	5.89 ± 0.29 a	8.69 ± 0.77 a	6.21 ± 0.39 a
**Flavones**	**67.15 ± 2.92 ab**	**61.54 ± 4.13 ab**	**79.26 ± 5.89 a**	**52.81 ± 5.15 b**	**53.03 ± 0.50 b**
Apigenin dihexoside	67.15 ± 2.92 ab	61.54 ± 4.13 ab	79.26 ± 5.89 a	52.81 ± 5.15 b	53.03 ± 0.50 b
**Flavonols**	**18.47 ± 0.59 a**	**16.64 ± 0.46 a**	**16.28 ± 2.64 a**	**12.93 ± 1.62 ab**	**8.65 ± 0.22 b**
Kaempferol glucuronyl dihexoside	0.32 ± 0.06 a	0.21 ± 0.03 a	0.31 ± 0.10 a	0.14 ± 0.03 a	0.29 ± 0.04 a
Kaempferol glucuronyl pentoside hexoside	0.08 ± 0.02 a	0.09 ± 0.02 a	0.13 ± 0.04 a	0.05 ± 0.01 a	0.04 ± 0.003 a
Kaempferol hexoside	1.05 ± 0.06 a	0.92 ± 0.03 a	0.90 ± 0.23 a	0.78 ± 0.16 a	0.59 ± 0.03 a
Kaempferol-3-rutinoside	1.98 ± 0.08 a	1.74 ± 0.03 a	1.95 ± 0.23 a	1.85 ± 0.18 a	1.45 ± 0.03 a
Kaempferol pentosyl hexoside	2.81 ± 0.15 a	2.65 ± 0.03 a	2.80 ± 0.40 a	2.46 ± 0.34 a	1.03 ± 0.07 b
Kaempferol rhamnosyl dihexoside	0.82 ± 0.08 a	0.81 ± 0.07 a	0.67 ± 0.13 a	0.62 ± 0.08 a	0.55 ± 0.02 a
Quercetin-3-xyloside	0.85 ± 0.12 a	0.71 ± 0.07 a	0.70 ± 0.30 a	0.50 ± 0.14 a	0.63 ± 0.13 a
Quercetin-3-arabinofuranoside	0.02 ± 0.001 a	0.01 ± 0.001 a	0.01 ± 0.003 a	0.01 ± 0.002 a	0.01 ± 0.001 a
Quercetin dihexoside	0.35 ± 0.02 a	0.29 ± 0.07 a	0.53 ± 0.19 a	0.28 ± 0.11 a	0.41 ± 0.04 a
Quercetin pentosyl hexoside 1	0.99 ± 0.03 a	0.84 ± 0.04 ab	0.86 ± 0.17 ab	0.62 ± 0.10 ab	0.55 ± 0.03 b
Quercetin pentosyl hexoside 2	4.69 ± 0.14 a	4.19 ± 0.09 a	3.92 ± 0.42 ab	2.97 ± 0.26 b	1.51 ± 0.05 c
Quercetin rhamnosyl hexoside	0.72 ± 0.02 a	0.62 ± 0.03 ab	0.63 ± 0.12 ab	0.45 ± 0.07 ab	0.40 ± 0.02 b
Quercetin-3-glucoside	3.81 ± 0.14 a	3.56 ± 0.06 a	2.89 ± 0.38 ab	2.19 ± 0.26 b	1.19 ± 0.06 c
**Stilbenes**	**18.68 ± 0.81 c**	**20.37 ± 2.91 c**	**34.62 ± 1.93 ab**	**37.33 ± 0.69 a**	**28.76 ± 1.69 b**
Piceatannol hexoside 1	5.71 ± 0.43 b	6. 47 ± 0.94 b	11. 51 ± 1.23 a	11.11 ± 0.59 a	9.92 ± 0.27 a
Piceatannol hexoside 2	6.99 ± 0.35 c	6. 71 ± 1.03 c	13.64 ± 0.36 b	16.98 ± 0.34 a	13.45 ± 0.80 b
Piceatannol hexoside 3	5.98 ± 0.37 b	7.19 ± 1.16 ab	9.47 ± 0.68 a	9.24 ± 0.17 a	5.38 ± 0.24 b
**Total polyphenolics**	**95.91 ± 2.74 b**	**105.87 ± 9.52 ab**	**124.84 ± 7.81 a**	**122.76 ± 3.15 a**	**93.71 ± 0.89 b**
